# Advances and Current Clinical Applications of Anterior Segment Optical Coherence Tomography Angiography

**DOI:** 10.3389/fmed.2021.721442

**Published:** 2021-11-23

**Authors:** Man Luo, Yiqing Li, Yehong Zhuo

**Affiliations:** State Key Laboratory of Ophthalmology, Zhongshan Ophthalmic Center, Sun Yat-sen University, Guangdong Provincial Key Laboratory of Ophthalmology and Visual Science, Guangzhou, China

**Keywords:** optical coherence tomography angiography, anterior segment, vascularization, clinical application, clinical diagnosis, efficacy evaluation

## Abstract

Optical coherence tomography angiography (OCTA) is the most relevant evolution based on optical coherence tomography (OCT). OCTA can present ocular vasculature, show detailed morphology for assessment, and quantify vessel parameters without intravenous dye agent. Research on the anterior segment OCTA (AS-OCTA) is only in its initial phase, and its advances in clinical diagnosis and treatment efficacy evaluations require a detailed comparison to traditional imaging methods. In this review of AS-OCTA, we summarize its technical features, imaging advances, current clinical applications in various eye diseases, as well as its limitations and potential future directions. AS-OCTA offers potential advantages in ophthalmic imaging, and with further development it could become a common tool in the near future.

## Introduction

Optical coherence tomography (OCT) provides a non-invasive and rapid way to image the eye *in vivo*. In the early 1990s, OCT was introduced to display retinal structures in cross-sections, such as the macula and optic nerve. Simultaneously, quantitative parameters of retina can also be detected, such as the peripapillary nerve fiber layer thickness, which is specifically identified in the development of open angle glaucoma (1, 2). With continuous development over the last 10 years, a new form of angiography, known as OCT angiography (OCTA), has been created (3). As the most relevant evolution of OCT, OCTA evaluates tissue from morphology to the study of functional components without intravenous injection of dye (4, 5). OCTA was designed for the posterior ocular segment, and has been widely used in age-related macular degeneration, high myopia, retinal vascular diseases, inflammatory diseases, and optic nerve diseases (2, 6, 7). OCTA allows better discrimination of the foveal avascular zone and parafoveal microvasculature than fluorescence angiography (FA), and can detect changes in lesions and early recurrence, even before the exudation detected on OCT (8–12). OCTA has improved identification of capillary non-perfusion, microaneurysms, and retinal ischemia, as well as delineating the foveal avascular zone (11, 13–22).

Most recently, a growing number of studies have been focusing on OCTA for anterior segment diseases. Anterior segment OCTA (AS-OCTA) provides a novel technique for acquiring anterior segment vasculature images (23). Although research on AS-OCTA is only in its initial phase, it has been gaining attention and recognition (7, 24, 25). Therefore, the application of AS-OCTA in clinical diagnosis and treatment needs to be summarized.

AS-OCTA offers several advantages over traditional angiography. Its non-invasive nature and rapid inspection time allow frequent repetition of tests, and avoids the potential risks of dye-based angiography (5). In addition, AS-OCTA provides cross-sectional high-resolution images of the vasculature and allows for analysis according to depth located on a specific axis (7, 26). It is also a more cost-effective method over conventional angiography that is more time-consumable and requires training-certified clinicians (24, 27).

Slit-lamp photography is the most common method for capturing anterior segment vasculature. However, the results of slit-lamp photography are easily influenced by corneal transparency and background iris vessels (24, 28). Dye-based techniques, including FA and indocyanine green angiography, are applied to assess vessels clinically (2, 24). However, dye administration is not suitable for some patients, and has various contraindications (2, 27–29). AS-OCT has advanced as a useful tool in providing rapid, non-contact evaluation of the anterior segment, and it takes a vital role in diagnosing and monitoring the effect of therapy in anterior segment diseases (30–32). However, AS-OCTA has developed the imaging technologies for increasing the speed and image resolution. Moreover, it offers the technology of evaluation of vascular flow (33). Furthermore, with the development of anti-angiogenic therapy, non-invasive techniques are needed for quantitative analysis in anterior segment vasculature (25). Because the OCTA systems are designed for posterior diseases, imaging the anterior segment requires putting on an adaptor lens (24, 29, 34). The quantitative index, including vessel density and blood flow, can monitor the effect of anti-vascular endothelial growth factor (VEGF) treatment, and guide treatment planning and prognostic evaluation (24, 25). Thus, an increasing role for AS-OCTA brings more attention and importance (35).

The aim of this review is to summarize the current applications of the AS-OCTA, including the diagnosis, therapy, and prognosis of detailed anterior diseases. In addition, the study discusses the limitations and potential future directions of AS-OCTA.

## Acquisition and Analysis of AS-OCTA

Different types of devices have been reported in AS-OCTA scanning (e.g., AngioVue, DRI Triton, and PLEX Elite 9000). The majority of the studies use adaptor lens, and manually adjust the equipment parameters F and Z for anterior segment imaging (F was adjusted to the minimum value and Z was close to the maximum) (36–41). During the whole scanning process, the external lens has been put near the eye, so we need to pay special attention during the inspection process to avoid damage (42). Some studies haven't used adaptor lens, applied the retina scanning procedure and manually adjusted the F value to the maximum value (39, 43). According to the anatomical structure of the eye, the diopter of posterior segment differs by 20D (diopters) from that of anterior segment (42). A self-designed lens holder was made by 3 Dimensions printing technology, which could place a 35D biconvex lens provided by Heidelberg at a suitable distance about 2 mm in front of the retinal lens to obtain an image (42). For the best image quality, the scanning area should be placed within the B scan range as far as possible. To avoid artifacts from the cornea and iris vessels, the scanning position can be placed flipping the zero-delay line and the image can be obtained “upside down” (39, 44). It is worth noting that the traditional image quality scoring standard and automatic image slicing function are not recommended in the current methods of obtaining AS-OCTA images (42).

## AS-OCTA Precise Diagnosis

### Conjunctiva

A chemical burn can lead to rapid and permanent denaturation to ocular surface (44). AS-OCTA was able to identify the normal vasculature in unaffected areas and areas of ischemia, similar in appearance to FA (45). Additionally, it can detect vascular flow though conjunctival edema and hemorrhages (46). In pterygium, AS-OCTA can detect more abundant vascularization in pterygium than in normal conjunctiva. In addition, these vessels are most obvious in the subepithelial connective tissue. The morphology of these vessels is newly formed, with smaller caliber, tortuous and branched shape, and rarely visible lumen (39). In the remaining connective tissue, the blood vessels have a regular diameter and morphology, very similar to normal conjunctive; their cavities are visible, sometimes dilated, and filled with blood (47). A study of scleral contact lens (ScCL) wearers demonstrates that repeated use of ScCL can cause vascular alterations in conjunctiva detected by AS-OCTA (48). Recent research shows that AS-OCTA works best on superficial and non-pigmented conjunctiva lesions, and malignant lesions always accompanied by deeper and larger perilesional vessels, which could be a significant marker of clinical diagnosis (49, 50).

### Cornea and Limbus

Combing structure and vascular information, OCTA can distinctly diagnose corneal pathologies (24–26). In addition, research suggests that AS-OCTA can show early corneal angiogenesis more clearly than slit-lamp photography (6, 24). OCTA can detect subtle abnormal blood vessels with corneal opacity, vascularization around the cornea or in front of the iris, which may not be detected by slit-lamp photography (6, 24). Both FA and indocyanine green angiography technologies only provide two-dimensional images, and dye leakage may hinder the analysis of deep blood vessels. OCTA allows simultaneous evaluation of the lesion structure and its related vessels in three-dimensions and at any predetermined depth (24). Severe corneal neovascularization (CoNV) always presents thicker, deeper, larger vessels on AS-OCTA (51). It has been noted that the extension and thickness of CoNV is related to the severity of limbal stem cell deficiency (LSCD) and best corrected visual acuity (BCVA) (52). In addition, Varma et al. (53) observed the conjunctivalization of the cornea caused by LSCD. The vascularization concentrated in superficial layer of peripheral cornea is the sign of LSCD, which can be differentiated from deeper vascularization caused by corneal inflammation.

### Ciliary Body and Iris

OCTA provides a non-invasive method for detecting small capillaries or microaneurysm flow, even at an early stage of neovascularization of the iris without leakage of dye, and only need little adjustments to the OCTA device (54, 55). Current assessments for neovascularization of the iris include slit lamp biomicroscopy, gonioscopy, FA, and indocyanine green angiography (56–58). Furthermore, conventional examinations may miss the very fine neovascularization of the iris (59, 60). Moreover, the assessment of neovascularization of the iris always needs a non-dilated pupil using dye-based angiography; thus, it is not possible to perform FA and indocyanine green angiography of the iris and retina at the same time, since most cases are caused by ischemic retinopathy (58).

The inflammation in the anterior uveitis always appears primarily on the iris and the ciliary body (3). During the active stage, the vessel in the iris dilates visibly. However, assessment of that presence is limited to previous examination, like fluorescein angiography (59). The reason is that the capillaries are non-fenestrated in the iris, and the leakage caused by inflammation cannot be shown by fluorescein angiography (59, 61). Since it is dye-free technology, AS-OCTA may provide more than a just promising method for assessing vascular anomalies in active anterior segment inflammation and para-inflammatory conditions. It might also be a safe and more precise method for investigating the pathophysiology of inflammation and the preclinical therapeutic strategies for uveitis (39, 55). Iris cysts appears with no intrinsic vascularity detected by AS-OCTA, and this could become a key imaging feature to distinguish iris cysts from other iris lesions (62).

## AS-OCTA for Efficacy Follow-Up

### Conjunctiva

The evaluation of limbal epithelial stem cells is related to the severity of the conjunctival chemical burn, therefore a reliable and objective assessment of limbal non-perfusion could make prognosis more accurate (63–65). The range of severe limbal ischemia determined by AS-OCTA represented the severity of LSCD (46). The correlation between AS-OCTA prognosis and visual outcome at 3 months supersedes that of FA. AS-OCTA could also be used for longitudinal clinical estimation of recovery and reperfusion (46). En face OCTA can potentially assess the deeper intrascleral vessels, and index the more severe damage that can leads to iris atrophy, cataract, secondary ocular hypertension, or hypotony (46).

Trabeculectomy remains the common surgical procedure for glaucoma when other treatments have failed to control intraocular pressure (66). Fibrosis in the filtering bleb area plays a crucial role in failure of trabeculectomy (67). Hence, an early and objective means of detecting bleb vascularity to minimize fibrosis and lead to better outcomes is important. The Indiana Bleb Appearance Grading Scale and Moorfields Bleb Grading System are clinically used, but these grading systems are quite subjective (68–70). The development in surgical area vascularity and tortuous vessels on bleb are signs of bleb failure (68, 70, 71). AS-OCTA could be applied to address the early evaluation of bleb, which plays a crucial role in discovering the failure of trabeculectomy (37, 38, 72–74). Bleb vascularity can be examined sensitively; a previous study showed a correlation between the outcome of AS-OCTA and the outcome of previous grading systems (75). AS-OCTA could potentially be used in evaluating vessel parameters pre- and post-trabeculectomy (75). In a recent study, vascularization of the filtering bleb assessed by AS-OCTA at 1-month post-trabeculectomy could predict surgery effects at 6 months post-treatment and provided timely post-surgery intervention for a more successful outcome from the trabeculectomy (37). A study focused on eyedrops finds that the lowering effect of IOP is associated with a lower baseline vessel density detected by AS-OCTA (76).

### Cornea and Limbus

AS-OCTA provides rapid processing and objective assessment of areas for corneal vessel invasion (77). OCTA is also useful for monitoring vascular changes before and after treatment. Furthermore, AS-OCTA allows estimations of the range and the depth of vascularization and fibrosis, such as the penetration of corneal abnormal vessels. This helps in pre-surgery planning, and monitoring of corneal vascularization after surgeries such as keratoplasty, pterygium surgery, and fine-needle diathermy (FND) combined with subconjunctival Ranibizumab (24–26, 78). In patients who received various treatments after graft rejection, AS-OCTA was able to find a significant decrease in the vascularized area after 3 months of treatment, which is consistent with color photographs (79).

### Ciliary Body and Iris

AS-OCTA is not only useful for screening, but also for following-up and assessment of treatments, such as laser or anti-VEGF therapy for neovascularization of the iris. Roberts et al. (55) showed that AS-OCTA provided a new stage for detecting neovascularization of the iris, which is regressed and clinically invisible. Additionally, en face OCTA can non-invasively show the persistent vessels by imaging blood flow after treatment. In the future, longitudinal follow-up will be indispensable for identifying OCTA markers for regression before and after therapy (37).

To better monitor the response to the treatment in clinical trials of anterior uveitis, efforts have been made utilizing multi-modal imaging to objectively measure ocular inflammation, for better patient monitoring and response measurement in clinical studies (80, 81). OCTA provides a superior objective measurement for showing the capillaries. Pichi et al. (82) showed that the dilatation and increased blood flow of an iris with acute anterior uveitis in OCTA could be recognized and quantified by AS-OCTA. This means that the iris vascular caliber might be a good index for quantifying the presence of inflammation and monitoring response to treatment. Choi et al. (83) compared the vessels caliber at different time points, whereas in the current study, an objective quantification was employed. A case of chronic retinal necrosis (CRN) finds that AS-OCTA can detect non-perfusion area of iris, and the reperfusion during the proper treatment, which could make an accurate follow-up of the progression (84).

## Current Limitation and Future Directions

Although AS-OCTA has many advantages for clinical applications, its limitations need to be addressed in future AS-OCTA studies. First, the OCTA device and software were designed for the posterior segment; imaging the anterior segment requires putting on an adaptor lens, which may yield lower quality images and inaccurate parameter values. A specific algorithm for anterior imaging with automated adjustment is necessary to improve and standardize the scan quality, such as the automation of focus and scaling (68, 85). Secondly, pupil size might affect vasculature caliber and morphological characteristics (39). Although studies have chosen similar lighting conditions, pupil size changes are unavoidable. The need to control the effect of environmental factors that can influence the vasculature in anterior segment should not be ignored; quantitatively reproducible measurements are needed to solve the problem. Thirdly, the conventional technique of OCTA lacks a sequential image matching function, which makes it hard to fix the same area of interest and reduce motion artifacts caused by severely decreased visual acuity. Thus, an eye-tracking system and sequential image matching system need to be incorporated (24, 85).

Furthermore, the restriction of the scanning field and signal dropout does not support high-quality imaging of both the perimeter part and the central part of region in interest. AS-OCTA evaluates a limited area and depth compared to AS-OCT, which is designed for the external eye. To meet this challenge, AS-OCT with angiography technology needs to be developed to help diagnosis and assessment of anterior segment diseases. Moreover, a wide field swept-source OCTA, which is operated at longer wavelengths, might be able to solve this issue (55, 77). Currently, wide-field swept-source OCTA provides a wider-angle image for detecting diabetic retinopathy lesions, such as 12 × 12 mm or even 15 × 9 mm using the Montage technique (86). What's more, wide-field swept-source OCTA shows huge prospects for anterior segment imaging due to its sufficient scanning range and high sensitivity in detecting structures.

## Conclusion

The imaging methods of AS-OCTA described here provide a rapid and non-invasive tool for specialized imaging. The technique allows detection of vessel diameter, density, and overall appearance, and provides powerful methods for helping clinical prognoses, diagnoses, and treatment outcomes (Table 1). Moreover, the challenges in AS-OCTA that were presented here should encourage the improvement of future techniques.

**Table 1:** Advantages and applications of AS-OCTA.

	**Precise diagnosis**	**Efficacy evaluation**
Conjunctiva	Identification of abnormal vasculature areas and conjunctival lesions	Longitudinal estimation of reperfusion
	Detection of deeper intrascleral vessels	Early evaluation of blood flow density on filtering bleb after trabeculectomy
	Assessment of limbal epithelial stem cell	
Cornea and limbus	Detection of early corneal angiogenesis	Preoperative surgical planning by evaluating the depth and area of feeder vessel
	Detection of subtle abnormal vessels with corneal opacity	Repeatable evaluation of treatment effect by vascular progression and regression
	3D imaging of cornea layers	Better observation of corneal graft rejection
	Scan of predetermined depth of cornea	
Ciliary body and iris	Early detection of small capillaries and microaneurysms flow	Evaluation of regressed neovascularization of the iris during anti-VEGF therapy
	Assessment of vascular anomalies in anterior uveitis	Exposure of persistent neovascularization
	Identification of iris lesions	

*VEGF, vascular endothelial growth factor; 3D, three dimensions*.

## Author Contributions

ML, YL, and YZ wrote and edited the manuscript. All authors contributed to the article and approved the submitted version.

## Funding

This work was supported by the National Natural Science Foundation of China (81870657, 81870658), the Natural Science Foundation of Guangdong Province (2018A030313049), the Medical Scientific Research Foundation of Guangdong Province (A2018052), and the Fundamental Research Funds for the Youth Scholars of Sun Yat-sen University (18ykpy32).

## Conflict of Interest

The authors declare that the research was conducted in the absence of any commercial or financial relationships that could be construed as a potential conflict of interest.

## Publisher's Note

All claims expressed in this article are solely those of the authors and do not necessarily represent those of their affiliated organizations, or those of the publisher, the editors and the reviewers. Any product that may be evaluated in this article, or claim that may be made by its manufacturer, is not guaranteed or endorsed by the publisher.

## References

[1] Koustenis A Jr Harris A Gross J Januleviciene I Shah A Siesky B. Optical coherence tomography angiography: an overview of the technology and an assessment of applications for clinical research. Br J Ophthalmol. (2017) 101:16–20. 10.1136/bjophthalmol-2016-309389 27707691

[2] Brunner M Romano V Steger B Vinciguerra R Lawman S Williams B. Imaging of corneal neovascularization: optical coherence tomography angiography and fluorescence angiography. Invest Ophthalmol Vis Sci. (2018) 59:1263–9. 10.1167/iovs.17-22035 29625447

[3] Spaide RF Fujimoto JG Waheed NK Sadda SR Staurenghi G. Optical coherence tomography angiography. Prog Retin Eye Res. (2018) 64:1–55. 10.1016/j.preteyeres.2017.11.003 29229445 PMC6404988

[4] Spaide RF. Volume-rendered optical coherence tomography of diabetic retinopathy pilot study. Am J Ophthalmol. (2015) 160:1200–10. 10.1016/j.ajo.2015.09.010 26384548

[5] Spaide RF Fujimoto JG Waheed NK. Optical coherence tomography angiography. Retina. (2015) 35:2161–2. 10.1097/IAE.0000000000000881 26502006 PMC4710360

[6] Oie Y Nishida K. Evaluation of corneal neovascularization using optical coherence tomography angiography in patients with limbal stem cell deficiency. Cornea. (2017) 36:S72–5. 10.1097/ICO.0000000000001382 28922332

[7] Tan ACS Tan GS Denniston AK Keane PA Ang M Milea D. An overview of the clinical applications of optical coherence tomography angiography. Eye. (2018) 32:262–86. 10.1038/eye.2017.181 28885606 PMC5811700

[8] Kuehlewein L Bansal M Lenis TL Iafe NA Sadda SR Bonin MA. Optical coherence tomography angiography of type 1 neovascularization in age-related macular degeneration. Am J Ophthalmol. (2015) 160:739–48. 10.1016/j.ajo.2015.06.030 26164826

[9] Inoue M Jung JJ Balaratnasingam C Dansingani KK Dhrami-Gavazi E Suzuki M. A comparison between optical coherence tomography angiography and fluorescein angiography for the imaging of type 1 neovascularization. Investig Ophthalmol Vis Sci. (2016) 57:314–323. 10.1167/iovs.15-18900 27409488

[10] Veronese C Maiolo C Morara M Armstrong GW Ciardella AP. Optical coherence tomography angiography to assess pigment epithelial detachment. Retina. (2016) 36:645–50. 10.1097/IAE.0000000000000914 26906495

[11] Soares M Neves C Marques IP Pires I Schwartz C Costa MA. Comparison of diabetic retinopathy classification using fluorescein angiography and optical coherence tomography angiography. Br J Ophthalmol. (2017) 101:62–8. 10.1136/bjophthalmol-2016-309424 27927677

[12] Tan ACS Dansingani KK Yannuzzi LA Sarraf D Freund KB. Type 3 Neovascularization imaged with cross-sectional and en face optical coherence tomography angiography. Retina. (2017) 37:234–46. 10.1097/IAE.0000000000001343 27749497

[13] de Castro-Abeger AH de Carlo TE Duker JS Baumal CR. Optical coherence tomography angiography compared to fluorescein angiography in branch retinal artery occlusion. Ophthalmic Surg Lasers Imaging Retina. (2015) 46:1052–4. 10.3928/23258160-20151027-12 26599250

[14] Kashani AH Lee SY Moshfeghi A Durbin MK Puliafito CA. Optical coherence tomography angiography of retinal venous occlusion. Retina. (2015) 35:2323–31. 10.1097/IAE.0000000000000811 26457395

[15] Cardoso JN Keane PA Sim DA Bradley P Agrawal R Addison PK. Systematic evaluation of optical coherence tomography angiography in retinal vein occlusion. Am J Ophthalmol. (2016) 163:93–107. 10.1016/j.ajo.2015.11.025 26621685

[16] Coscas F Glacet-Bernard A Miere A Caillaux V Uzzan J Lupidi M. Optical coherence tomography angiography in retinal vein occlusion: evaluation of superficial and deep capillary plexa. Am J Ophthalmol. (2016) 161:160–71. 10.1016/j.ajo.2015.10.008 26476211

[17] Suzuki N Hirano Y Yoshida M Tomiyasu T Uemura A Yasukawa T. (2016). Microvascular abnormalities on optical coherence tomography angiography in macular edema associated with branch retinal vein occlusion. Am J Ophthalmol. 161:126–32e1. 10.1016/j.ajo.2015.09.038 26454243

[18] Dimitrova G Chihara E Takahashi H Amano H Okazaki K. Quantitative retinal optical coherence tomography angiography in patients with diabetes without diabetic retinopathy. Investig Ophthalmol Vis Sci. (2017) 58:190–6. 10.1167/iovs.16-20531 28114579

[19] Iafe N Falavarjani KG Hubschman JP Tsui I Sadda SR Sarraf D. (2017). Optical coherence tomography angiography analysis of the foveal avascular zone and macular vessel density after anti-VEGF therapy in eyes with diabetic macular edema and retinal vein occlusion. Investig Ophthalmol Vis Sci. 58:30–4. 10.1167/iovs.16-20579 28114569

[20] Krawitz BD Mo S Geyman LS Agemy SA Scripsema NK Garcia PM. Acircularity index and axis ratio of the foveal avascular zone in diabetic eyes and healthy controls measured by optical coherence tomography angiography. Vision Res. (2017) 139:177–86. 10.1016/j.visres.2016.09.019 28212983

[21] Singh A Agarwal A Mahajan S Karkhur S Singh R Bansal R. Morphological differences between optic disc collaterals and neovascularization on optical coherence tomography angiography. Graefes Arch Clin Exp Ophthalmol. (2017) 255:753–9. 10.1007/s00417-016-3565-x 27942950

[22] Kadomoto S Muraoka Y Ooto S Miwa Y Iida Y Suzuma K. Evaluation of macular ischemia in eyes with branch retinal vein occlusion an optical coherence tomography angiography study. Retina. (2018) 38:272–82. 10.1097/IAE.0000000000001541 28221256

[23] Ang M Devarajan K Das S Stanzel T Tan A Girard M. Comparison of anterior segment optical coherence tomography angiography systems for corneal vascularisation. Br J Ophthalmol. (2018) 102:873–7. 10.1136/bjophthalmol-2017-311072 28939690

[24] Ang M Sim DA Keane PA Sng CCA Egan CA Tufail A. Optical coherence tomography angiography for anterior segment vasculature imaging. Ophthalmology. (2015) 122:1740–7. 10.1016/j.ophtha.2015.05.017 26088621

[25] Ang M Cai YJ Shahipasand S Sim DA Keane PA Sng CCA. En face optical coherence tomography angiography for corneal neovascularisation. Br J Ophthalmol. (2016) 100:616–21. 10.1136/bjophthalmol-2015-307338 26311064

[26] Ang M Tan ACS Cheung CMG Keane PA Dolz-Marco R Sng CCA. Optical coherence tomography angiography: a review of current and future clinical applications. Graefes Arch Clin Exp Ophthalmol. (2018) 256:237–45. 10.1007/s00417-017-3896-2 29318383

[27] Cai YJ del Barrio JLA Wilkins MR Ang M. (2017). Serial optical coherence tomography angiography for corneal vascularization. Graefes Arch Clin Exp Ophthalmol. 255:135–9. 10.1007/s00417-016-3505-9 27722920

[28] Stenzel TP Devarajan K Lwin NC Yam GH Schmetterer L Mehta JS. Comparison of optical coherence tomography angiography to indocyanine green angiography and slit lamp photography for corneal vascularization in an animal model. Sci Rep. (2018) 8:11493. 10.1038/s41598-018-29752-5 30065317 PMC6068177

[29] Ang M Cai YJ MacPhee B Sim DA Keane PA Sng CCA. Optical coherence tomography angiography and indocyanine green angiography for corneal vascularisation. Br J Ophthalmol. (2016) 100:1557–63. 10.1136/bjophthalmol-2015-307706 26823396

[30] Ang M Chong W Tay WT Yuen L Wong TY He MG. Anterior segment optical coherence tomography study of the cornea and anterior segment in adult ethnic south asian indian eyes. Investig Ophthalmol Vis Sci. (2012) 53:120–5. 10.1167/iovs.11-8386 22025574

[31] Ang M Chong W Huang HQ Tay WT Wong TY He MG. (2013). Comparison of anterior segment optical tomography parameters measured using a semi-automatic software to standard clinical instruments. PLoS ONE. 8:e65559. 10.1371/journal.pone.0065559 23750265 PMC3672133

[32] Venkateswaran N Galor A Wang JH Karp CL. Optical coherence tomography for ocular surface and corneal diseases: a review. Eye Vis. (2018) 5:13. 10.1186/s40662-018-0107-0 29942817 PMC5996489

[33] Spaide RF Klancnik J Cooney MJ. Retinal vascular layers imaged by fluorescein angiography and optical coherence tomography angiography. JAMA Ophthalmol. (2015) 133:45–50. 10.1001/jamaophthalmol.2014.3616 25317632

[34] Ang M Baskaran M Werkmeister RM Chua J Schmidl D dos Santos VA. Anterior segment optical coherence tomography. Prog Retin Eye Res. (2018) 66:132–56. 10.1016/j.preteyeres.2018.04.002 29635068

[35] Lee WD Devarajan K Chua J Schmetterer L Mehta JS Ang M. Optical coherence tomography angiography for the anterior segment. Eye Vis. (2019) 6:4. 10.1186/s40662-019-0129-2 30775387 PMC6357412

[36] Akagi T Uji A Huang AS Weinreb RN Yamada T Miyata M. Conjunctival and intrascleral vasculatures assessed using anterior segment optical coherence tomography angiography in normal eyes. Am J Ophthalmol. (2018) 196:1–9. 10.1016/j.ajo.2018.08.009 30099035 PMC6284828

[37] Yin X Cai QH Song R He XF Lu PR. Relationship between filtering bleb vascularization and surgical outcomes after trabeculectomy: an optical coherence tomography angiography study. Graefes Arch Clin Exp Ophthalmol. (2018) 256:2399–405. 10.1007/s00417-018-4136-0 30209568

[38] Hayek S Labbe A Brasnu E Hamard P Baudouin C. Optical coherence tomography angiography evaluation of conjunctival vessels during filtering surgery. Transl Vis Sci Technol. (2019) 8:4. 10.1167/tvst.8.4.4 31293822 PMC6613592

[39] Pichi F Roberts P Neri P. The broad spectrum of application of optical coherence tomography angiography to the anterior segment of the eye in inflammatory conditions: a review of the literature. J Ophthalmic Inflamm Infect. (2019) 9:18. 10.1186/s12348-019-0184-9 31485882 PMC6726732

[40] Seo JH Lee Y Shin JH Kim YA Park KH. Comparison of conjunctival vascularity changes using optical coherence tomography angiography after trabeculectomy and phacotrabeculectomy. Graefes Arch Clin Exp Ophthalmol. (2019) 257:2239–55. 10.1007/s00417-019-04412-0 31292762

[41] Siddiqui Y Yin J. Anterior segment applications of optical coherence tomography angiography. Semin Ophthalmol. (2019) 34:264–9. 10.1080/08820538.2019.1620805 31188047

[42] Ayres M Smallwood R Brooks AM Chan E Fagan X. Anterior segment optical coherence tomography angiography. J Vis Commun Med. (2019) 42:153–7. 10.1080/17453054.2019.1631122 31402723

[43] Mastropasqua R Brescia L Di Antonio L Guarini D Giattini D Zuppardi E. Angiographic biomarkers of filtering bleb function after XEN gel implantation for glaucoma: an optical coherence tomography-angiography study. Acta Ophthalmol. (2020) 98:e761–7. 10.1111/aos.14371 32020755

[44] Wagoner MD. Chemical injuries of the eye: current concepts in pathophysiology and therapy. Surv Ophthalmol. (1997) 41:275–313. 10.1016/S0039-6257(96)00007-0 9104767

[45] Kuckelkorn R Remky A Wolf S Reim M Redbrake C. Video fluorescein angiography of the anterior eye segment in severe eye burns. Acta Ophthalmol Scand. (1997) 75:675–80. 10.1111/j.1600-0420.1997.tb00629.x 9527330

[46] Fung SSM Stewart RMK Dhallu SK Sim DA Keane PA Wilkins MR. Anterior segment optical coherence tomographic angiography assessment of acute chemical injury. Am J Ophthalmol. (2019) 205:165–74. 10.1016/j.ajo.2019.04.021 31078533

[47] Liu YC Devarajan K Tan TE Ang M Mehta JS. Optical coherence tomography angiography for evaluation of reperfusion after pterygium surgery. Am J Ophthalmol. (2019) 207:151–8. 10.1016/j.ajo.2019.04.003 30959005

[48] Jesus J Dias L Almeida I Costa T Chibante-Pedro J. Analysis of conjunctival vascular density in scleral contact lens wearers using optical coherence tomography angiography. Cont Lens Anterior Eye. (2021) 101403. 10.1016/j.clae.2020.12.066. [Epub ahead of print]. 33583731

[49] Binotti WW Mills H Nose RM Wu HK Duker JS Hamrah P. Anterior segment optical coherence tomography angiography in the assessment of ocular surface lesions. Ocul Surf. (2021) 22:86–93. 10.1016/j.jtos.2021.07.009 34333154 PMC11583202

[50] Brouwer NJ Marinkovic M Bleeker JC Luyten GPM Jager MJ. Anterior segment OCTA of melanocytic lesions of the conjunctiva and iris. Am J Ophthalmol. (2021) 222:137–47. 10.1016/j.ajo.2020.09.009 32926848

[51] Binotti WW Koseoglu ND Nose RM Kenyon KR Hamrah P. Novel parameters to assess the severity of corneal neovascularization using anterior segment optical coherence tomography angiography. Am J Ophthalmol. (2021) 222:206–17. 10.1016/j.ajo.2020.08.023 32822670 PMC11791782

[52] Binotti WW Nose RM Koseoglu ND Dieckmann GM Kenyon K Hamrah P. The utility of anterior segment optical coherence tomography angiography for the assessment of limbal stem cell deficiency. Ocul Surf. (2021) 19:94–103. 10.1016/j.jtos.2020.04.007 32335247

[53] Varma S Shanbhag SS Donthineni PR Mishra DK Singh V Basu S. High-resolution optical coherence tomography angiography characteristics of limbal stem cell deficiency. Diagnostics (Basel). (2021) 11:1130. 10.3390/diagnostics11061130 34205702 PMC8233779

[54] Gartner S Henkind P. Neovascularization of the iris (rubeosis iridis). Surv Ophthalmol. (1978) 22:291–312. 10.1016/0039-6257(78)90175-3 349748

[55] Roberts PK Goldstein DA Fawzi AA. Anterior segment optical coherence tomography angiography for identification of iris vasculature and staging of iris neovascularization: a pilot study. Curr Eye Res. (2017) 42:1136–42. 10.1080/02713683.2017.1293113 28441067 PMC5753407

[56] Parodi MB Bondel E Russo D Ravalico G. Iris indocyanine green videoangiography in diabetic iridopathy. Br J Ophthalmol. (1996) 80:416–9. 10.1136/bjo.80.5.416 8695562 PMC505492

[57] Brancato R Bandello F Lattanzio R. Iris fluorescein angiography in clinical practice. Surv Ophthalmol. (1997) 42:41–70. 10.1016/S0039-6257(97)84042-8 9265702

[58] Sivak-Callcott JA O'Day DM Gass JD Tsai JC. Evidence-based recommendations for the diagnosis and treatment of neovascular glaucoma. Ophthalmology. (2001) 108:1767–76. 10.1016/S0161-6420(01)00775-8 11581047

[59] Bandello F Brancato R Lattanzio R Falcomata B Malegori A. Biomicroscopy versus fluorescein angiography of the iris in the detection of diabetic iridopathy. Graefes Arch Clin Exp Ophthalmol. (1993) 231:444–8. 10.1007/BF02044229 8224942

[60] Li ZQ Zhou XX Lin S Li JL Wu JG. Angiography reveals early hiding iris neovascularization after ischemic CRVO. Int J Ophthalmol. (2013) 6:253–4. 10.3980/j.issn.2222-3959.2013.02.28 23638433 PMC3633771

[61] Danzig CJ Shields CL Mashayekhi A Ehya H Manquez ME Shields JA. Fluorescein angiography of iris juvenile xanthogranuloma. J Pediatr Ophthalmol Strabismus. (2008) 45:110–2. 10.3928/01913913-20080301-03 18404959

[62] Kose HC Gunduz K Hosal MB. Iris Cysts: Clinical features, imaging findings, and treatment results. Turk J Ophthalmol. (2020) 50:31–6. 10.4274/tjo.galenos.2019.20633 32167261 PMC7086092

[63] Roper-Hall MJ. Thermal and chemical burns. Trans Ophthalmol Soc U K. (1965) 85:631–53. 5227208

[64] Dua HS King AJ Joseph A. A new classification of ocular surface burns. Br J Ophthalmol. (2001) 85:1379–83. 10.1136/bjo.85.11.1379 11673310 PMC1723789

[65] Bagley DM Casterton PL Dressler WE Edelhauser HF Kruszewski FH McCulley JP. Proposed new classification scheme for chemical injury to the human eye. Regul Toxicol Pharmacol. (2006) 45:206–13. 10.1016/j.yrtph.2006.04.005 16764976

[66] Cairns JE. Trabeculectomy. Preliminary report of a new method. Am J Ophthalmol. (1968) 66:673–9. 10.1016/0002-9394(68)91288-9 4891876

[67] Landers J Martin K Sarkies N Bourne R Watson P. A Twenty-year follow-up study of trabeculectomy: risk factors and outcomes. Ophthalmology. (2012) 119:694–702. 10.1016/j.ophtha.2011.09.043 22196977

[68] Cantor LB Mantravadi A WuDunn D Swamynathan K Cortes A. Morphologic classification of filtering Blebs after glaucoma filtration surgery: The Indiana bleb appearance grading scale. J Glaucoma. (2003) 12:266–71. 10.1097/00061198-200306000-00015 12782847

[69] Crowston JG Kirwan JF Wells A Kennedy C Murdoch IE. Evaluating clinical signs in trabeculectomized eyes. Eye. (2004) 18:299–303. 10.1038/sj.eye.6700638 15004581

[70] Wells AP Crowston JG Marks J Kirwan JF Smith G Clarke JCK. A pilot study of a system for grading of drainage blebs after glaucoma surgery. J Glaucoma. (2004) 13:454–60. 10.1097/00061198-200412000-00005 15534469

[71] Picht G Grehn F. Classification of filtering blebs in trabeculectomy: biomicroscopy and functionality. Curr Opin Ophthalmol. (1998) 9:2–8. 10.1097/00055735-199804000-00002 10180508

[72] Leung CK Yick DW Kwong YY Li FC Leung DY Mohamed S. Analysis of bleb morphology after trabeculectomy with Visante anterior segment optical coherence tomography. Br J Ophthalmol. (2007) 91:340–4. 10.1136/bjo.2006.100321 17005548 PMC1857643

[73] Jia YL Wei E Wang XG Zhang XB Morrison JC Parikh M. Optical coherence tomography angiography of optic disc perfusion in glaucoma. Ophthalmology. (2014) 121:1322–32. 10.1016/j.ophtha.2014.01.021 24629312 PMC4082728

[74] Watson P Romano A. The impact of new methods of investigation and treatment on the understanding of the pathology of scleral inflammation. Eye. (2014) 28:915–30. 10.1038/eye.2014.110 24875228 PMC4135249

[75] Seo JH Kim YA Park KH Lee Y. Evaluation of functional filtering bleb using optical coherence tomography angiography. Transl Vis Sci Technol. (2019) 8:14. 10.1167/tvst.8.3.14 31110915 PMC6504203

[76] Akagi T Okamoto Y Kameda T Suda K Nakanishi H Miyake M. Short-term effects of different types of anti-glaucoma eyedrop on the sclero-conjunctival vasculature assessed using anterior segment OCTA in normal human eyes: a pilot study. J Clin Med. (2020) 9:4016. 10.3390/jcm9124016 33322580 PMC7764657

[77] Kirwan RP Zheng YL Tey A Anijeet D Sueke H Kaye SB. Quantifying changes in corneal neovascularization using fluorescein and indocyanine green angiography. Am J Ophthalmol. (2012) 154:850–8. 10.1016/j.ajo.2012.04.021 22840481

[78] Foo VHX Ke M Tan CQL Schmetterer L Mehta JS Ang M. anterior segment optical coherence tomography angiography assessment of corneal vascularisation after combined fine-needle diathermy with subconjunctival ranibizumab: a pilot study. Adv Ther. (2021) 38:4333–43. 10.1007/s12325-021-01849-w 34241779

[79] Harun S Srinivasan S Hollingworth K Batterbury M Kaye S. Modification of classification of ocular chemical injuries. Br J Ophthalmol. (2004) 88:1353–4. 10.1136/bjo.2004.046797/047308 15377570 PMC1772368

[80] Li Y Lowder C Zhang XB Huang D. Anterior chamber cell grading by optical coherence tomography. Investig Ophthalmol Vis Sci. (2013) 54:258–65. 10.1167/iovs.12-10477 23249705 PMC3544530

[81] Sharma S Lowder CY Vasanji A Baynes K Kaiser PK Srivastava SK. Automated analysis of anterior chamber inflammation by spectral-domain optical coherence tomography. Ophthalmology. (2015) 122:1464–70. 10.1016/j.ophtha.2015.02.032 25846846

[82] Pichi F Sarraf D Arepalli S Lowder CY Cunningham ET Jr Neri P. (2017). The application of optical coherence tomography angiography in uveitis and inflammatory eye diseases. Prog Retin Eye Res. 59:178–201. 10.1016/j.preteyeres.2017.04.005 28465249

[83] Choi WJ Pepple KL Zhi ZW Wang RK. Optical coherence tomography based microangiography for quantitative monitoring of structural and vascular changes in a rat model of acute uveitis *in vivo*: a preliminary study. J Biomed Optics. (2015) 20:016015. 10.1117/1.JBO.20.1.016015 25594627 PMC4296737

[84] Zicarelli F Parrulli S Torre A Oldani M Invernizzi A. Optical coherence tomography angiography findings of iris ischemia and reperfusion in cytomegalovirus panuveitis. Ocul Immunol Inflamm. 10.1080/09273948.2021.1916041 34114921

[85] Akagi T Uji A Okamoto Y Suda K Kameda T Nakanishi H. Anterior segment optical coherence tomography angiography imaging of conjunctiva and intrasclera in treated primary open-angle glaucoma. Am J Ophthalmol. (2019) 208:313–22. 10.1016/j.ajo.2019.05.008 31102577

[86] Zhu Y Cui Y Wang JC Lu YF Zeng R Katz R. Different scan protocols affect the detection rates of diabetic retinopathy lesions by wide-field swept-source optical coherence tomography angiography. Am J Ophthalmol. (2020) 215:72–80. 10.1016/j.ajo.2020.03.004 32205122

